# Reviewer acknowledgement 2012

**DOI:** 10.1186/1746-160X-9-6

**Published:** 2013-02-19

**Authors:** Thomas Stamm, Ulrich Meyer, Hans Peter Wiesmann

**Affiliations:** 1 University of Münster, Münster, Germany; 2 University of Düsseldorf, Düsseldorf, Germany; 3 Technische Universität Dresden, Dresden, Germany

## Abstract

**Contributing reviewers:**

The Editors of *Head & Face Medicine* would like to thank all our reviewers who have contributed to the journal in Volume 8 (2012).

## 

Shahram Agah

Iran

Igor Aurer

Croatia

Salman Baig

Pakistan

Zaid Baqain

Jordan

Arnulf Baumann

Austria

Farzad Borumandi

Austria

Luca Campana

Italy

Paolo Castellucci

Italy

Ales Celar

Austria

Lucia Cevidanes

United States of America

Bernard Cohen

United States of America

Ian Cooper

United Kingdom

Adriano Crismani

Austria

Gholamreza Danesh

Germany

Jan de Lange

Netherlands

Willian de Melo

Brazil

Tim Desmet

Belgium

Charles Diaper

United Kingdom

Xi Ding

China

Márcio Diniz-Freitas

Spain

Marco Donto

United States of America

Murat Durdu

Turkey

Theodore Eliades

Switzerland

Ijlal Erturan

Turkey

Seyed-Mohammad Fereshtehnejad

Sweden

Tadashi Fujita

Japan

Anne George

United States of America

Anita Gortho

India

Dan Grauer

United States of America

Mohammed Grawish

Egypt

Maria Grosheva

Germany

Santosh Kumar Gudi

India

Selim Güleryüz

Germany

Pierre Haen

France

Trent Hargens

United States of America

Esam Helboub

Yemen

Alexander Hemprich

Germany

Johannes Hidding

Germany

Yoshihito Ishihara

Japan

David E Jaramillo

United States of America

Ramzi Jeddi

Tunisia

Manish Juneja

India

Simone Junqueira

Brazil

Luis Junquera

Spain

Hiroshi Kaji

Japan

Solveiga Kelbauskiene

United Kingdom

Yusuke Kiyoura

Japan

Johannes Kleinheinz

Germany

Cornelia Kober

Germany

Norio Kondo

Japan

Michel Kossowski

France

Adorján Kovács

Germany

Stefan Kranz

Germany

Eike Krause

Germany

Elena Krieger

Germany

Derek Lacey

Australia

Friedrich Lampert

Germany

José Angelo Lauletta Lindoso

Brazil

Mark Lauren

United States of America

Pierre Layrolle

France

Carsten Lippold

Germany

Tian-Run Liu

China

Xinqiang Liu

China

Matilde Mia Lonka

Denmark

Joanna Maclean

Canada

Evelina Maines

Italy

Seicho Makihira

Japan

Serge Marbacher

Switzerland

Niall Mcleod

United Kingdom

Shabnum Meer

South Africa

Marcello Melis

Italy

Luis Molfetta

France

Marco Mozzati

Italy

Marcella Nebbioso

Italy

Artor Niccoli Asabella

Italy

Masahiro Nishimura

Japan

Almudena Nuno-Gonzalez

Spain

Won-Suk Oh

United States of America

Thais Marchini Oliveira

Brazil

Cengiz Özcan

Turkey

William Papaioannou

Greece

Tannamala Pavan Kumar

India

Petia Pechalova

Bulgaria

Jozsef Piffko

Hungary

Satapong Pisuitthanakan

Thailand

Edela Puricelli

Brazil

Yasser Ragab

Egypt

Mahesh Ramanan

Australia

Majeed Rana

Germany

Shiv Shankar Saha

India

Rajab Said

United Kingdom

Olcay Sakar

Turkey

Hazem Saleh

Egypt

Ulrich Schlagenhauf

Germany

Karl Andreas Schlegel

Germany

Udo Schumacher

Germany

Wesley Shankland

United States of America

Bernd W Sigusch

Germany

Armando Silvestrini Biavati

Italy

Frederich Syffold

France

Irene Tami-Maury

United States of America

Hans J Ten Donkelaar

Netherlands

Kobkan Thongprasom

Thailand

Paolo Tosco

Italy

Satoru Tsuiki

Japan

Ram Ballabh Upadhyay

India

Elena Varoni

Italy

Francesco Vergani

United Kingdom

Paolo Vescovi

Italy

Saman Warnakulasuriya

United Kingdom

Dieter Weingart

Germany

Dirk Wiechmann

Germany

Edward Wright

United States of America

Seiichi Yoshimoto

Japan

